# Critical ion velocities for nanostructure formation on metal surfaces by slow highly charged ions at arbitrary incidence angles

**DOI:** 10.1038/s41598-025-17634-6

**Published:** 2025-10-02

**Authors:** Milena D. Majkić, Nataša N. Nedeljković, Dimitrije P. Majkić, Dariusz Banaś, Ilona Stabrawa

**Affiliations:** 1 Faculty of Technical Sciences, University of Priština-Kosovska Mitrovica, Knjaza Miloša 7, Kosovska Mitrovica, 38220 Serbia; 2 Faculty of Physics, University of Belgrade, P.O. Box 368, Belgrade, 11001 Serbia; 3 School of Electrical and Computer Engineering, Academy of Technical and Art Applied Studies, Belgrade, 11000 Serbia; 4 Institute of Physics, Jan Kochanowski University, Uniwersytecka 7, 25-406 Kielce, Poland

**Keywords:** Highly charged ions, Metal surface, Incidence angle, Surface nanostructure, Velocity effect, Neutralization energy, Materials science, Nanoscience and technology, Physics

## Abstract

Surface nanostructuring of metal targets induced by irradiation with slow, highly charged ions at various incidence angles is investigated. We extend our recently developed cohesive energy model to analyze the influence of impact geometry. At low ion energies, both the neutralization energy and the deposited kinetic energy, resulting from processes occurring above and below the surface, contribute to changes in the material’s morphology. We define the critical ion velocity as a key parameter that characterizes the interplay between these two energy contributions and determines which mechanism predominantly drives nano-sized object formation. Based on the critical velocity definition, hillocks formation indicates that the neutralization energy is a dominant energy source, whereas craters formation implies that the deposited kinetic energy plays a significant role for a given collision geometry. The effect of the incidence angle on the critical ion velocity is examined in detail. The model predictions for angular effects are applied to a system consisting of slow, highly charged $\hbox {Xe}^{q+}$ ions interacting with a gold target.

## Introduction

The bombardment of solids by slow highly charged ions (HCI) or swift heavy ions (SHI) may lead to surface modification in the form of nano-sized features such as hillocks, craters, ring, caldera like structures and pits^1–5^. These modifications are influenced by various parameters, including the ion kinetic energy (velocity), potential energy (charge state)^1,5,6^, and impact geometry^2,7,8^.

Initial experiments with HCI demonstrated that, for a given material, typically only one type of nanostructure is formed^6^. For example, hillock-like structures have been observed following interactions between HCI and materials such as $\hbox {CaF}_2$, $\hbox {BaF}_2$, or $\hbox {Al}_2$ $\hbox {O}_3$^9–12^, whereas craters (pits) are generated on KBr, Si, PMMA, and KCl^1^ (see also references in therein). Recent studies, however, have demonstrated that by varying the ion charge and velocity, diverse nanostructures can be produced on the same material. Specifically, authors in^6^ showed that three distinct nanostructure types can form on LiF when the charge state (potential energies) of HCI is varied at a constant velocity of $v=0.176$ a.u. Similarly, it has been reported in^3,4^ that, altering the HCI’s kinetic energy leads to either hillocks or craters formation on a gold target.

Additionally, impact geometry, alongside ion energy and charge state, can play a significant role in determining the type of nanostructure formed. For instance, when SHI strike the surface perpendicularly, as in the cases of $\hbox {TiO}_2$ and $\hbox {CaF}_2$, conically shaped defective regions and hillocks have been observed^8,13^ (see also references therein). In contrast, irradiations under oblique incidence ($1^0$) can lead to the formation of grooves (e.g. in MgO^7^, CaF2^14^) or chains of hillocks, as observed in $\hbox {Al}_2$ $\hbox {O}_3$^7^. Similarly, the authors in^2^ reported that 260 keV $\hbox {Xe}^+$ ions incident at 70$^{0}$ relative to the surface normal produce craters on PMMA films, while no craters were seen under normal incidence.

So far, to the best of our knowledge, no investigation of the correlation between irradiation geometry and the shape of nanostructures created by HCI has been reported. However, the influence of incidence geometry on HCI-metal surface interactions has been explored through measurements of secondary electron emission. For instance, the angular dependence of total electron yields for $\hbox {Ar}^{q+}$ and $\hbox {Xe}^{q+}$ ions impinging on a clean single crystal Au(111) surface and on HOPG has been discussed in^15,16^, respectively. They observed, for a given charge state *q*, an increase in electron yield with decreasing incidence angle (relative to the surface plane). Furthermore, the influence of impact geometry on HCI-surface interaction process has been studied with regard to the electron energy loss of slow highly charged $\hbox {Ar}^{q+}$ ions grazing on the Al(111) single-crystal surface as well as tungsten surfaces, under various azimuthal angles^17,18^.

Comprehensive experimental investigations of the interaction mechanisms are crucial from a theoretical perspective, as a unified understanding of the nanostructure creation process remains lacking. Up to now, the proposed theoretical models of nanostructure formation, including the Coulomb explosion^19–21^, molecular dynamics simulations^22^ and inelastic thermal spike model^23,24^, have been primarily focused on insulator targets. To address the nanostructure formation on metallic surfaces, a two-step cohesive energy model (CEM)^25^ has been recently developed.

The first step of the model is devoted to the calculation of energies (neutralization and deposited kinetic energy). The neutralization energy (a fraction of potential energy deposited into the surface) arises from the cascade neutralization of the ion, a process that takes place above the surface. On the other hand, elastic collisions between the projectile and target atoms (nuclear stopping power), and inelastic collisions between the projectile and electrons in the target (electronic stopping power), contribute to the energy loss. For velocities characteristic for slow HCI-solid interactions, the nuclear stopping regime is a dominant one^3,26,27^. Both energies are essential for material nanostructuring, but one prevails giving a primarily contribution and governing the specific nanostructure formation^4,25,28^. To determine the dominant mechanism, we introduce a threshold in ion velocity, referred to as the critical ion velocity, at which the two contributions are equal. Below this threshold, the neutralization energy exceeds the deposited kinetic energy contribution, leading to the hillock formation^3,25,28^. On contrary, for ion velocities above the critical value, the deposited kinetic energy becomes a dominant factor, inducing the crater (ring) formation^4,5^.

The core concept of the second step of the model is that the total deposited energy leads to a change in the target’s cohesive energy, thereby inducing surface modification. Cohesive energy is a fundamental property of metallic bonding, defined as the energy required to completely decompose a solid into its constituent atoms.

In the present work, we extend the cohesive energy model (CEM) to study the influence of impact geometry on surface modification. In previous studies^4,25,28^, only normal incidence geometry was considered. Up to now, the irradiation geometry effect has been included in calculations of population probabilities^29^, final ion charge states (charge state of the ion in front of the surface), and neutralization energy^30^. To account for angular effects, we modify the micro staircase model (MSCM), based on the two-state vector model (TVM), to evaluate the neutralization energy. The deposited kinetic energy is calculated using the charge-dependent interaction potential model^28,31^. The fundamental aspects of slow HCI-solid interactions are described, with particular attention to processes occurring both above and below the surface. First, we analyse the final ion charges as a function of ion velocity for various incidence angles. This dependence influences both the neutralization energy and deposited kinetic energy. Specifically, as the incidence angle decreases, the final ion charges decrease, resulting in an increase in both the neutralization energy^30^ and kinetic energy loss. The interplay between these two energy contributions governs the shape of the formed nanostructure and is determined by the critical ion velocity. The critical ion velocity is found to be angle-dependent.

This article is organized as follows. In “Extension of CEM I to arbitrary geometry introduction” section, we extend the first step of the cohesive energy model (CEM I) to arbitrary collision geometries. In “Nanostructure shape under arbitrary collision geometry” section, we analyse the effect of the incidence angle on the shape of the resulting nanostructures. In “Critical ion velocity under arbitrary collision geometry” section, we examine how the incidence angle influences the critical ion velocity. The concluding remarks are given in “Concluding remarks” section.

Atomic units ($e^{{ 2}} = \hbar = m_{{e}} = 1$) will be used throughout the paper unless indicated otherwise.

## Extension of CEM I to arbitrary geometry introduction

### Cascade neutralization process above the surface under arbitrary collision geometry

**Fig. 1:**
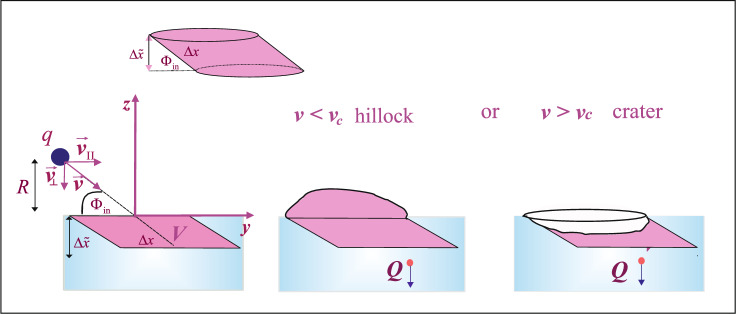
Schematic illustration of nanohillock and nanocrater formation induced by the impact of a slow, highly charged ion (HCI) on a solid surface at an arbitrary incidence angle.

We consider slow, highly charged $\hbox {Xe}^{q+}$ ions (with charge $q\gg 1$) impinging on a metal surface at an arbitrary angle of incidence $\Phi _{in}$ (measured relative to the surface plane, see Fig. 1. The ion velocities *v* range from low to moderate ($v \le 0.4$ a.u.), where $\textbf{v}= - v_{\perp }\textbf{e}_z + v_{\parallel }\textbf{e}_y$. The normal ion-surface distance *R* is related to the perpendicular velocity component via the relation $v_{\perp } = -dR/ dt$, see Fig. 1.

The cascade neutralization process that occurs above the surface is described by the scheme *q*→ *q*–1..→ *Q*(*R*)... → $Q_{{fin}}^{(q)}$, where *Q*(*R*) and $Q_{{fin}}^{(q)}$ denote the ion charge at intermediate ion-surface distance *R* and very vicinity of the surface, respectively. This process is described within the micro staircase model (MSCM)^29^, which accounts for the fine structure of the neutralization cascades. Within the MSCM framework, the ion neutralization of initial charge *q* proceeds through macro-steps, each consisting of smaller steps called micro-cascades. At each macro step of the cascade, electrons are captured into high-lying projectile states (Rydberg states). These states decay to the (almost) ground state through radiative processes and when the ion approaches the surface-via Auger-type processes accompanied by secondary electron emission, in interplay with the population dynamics described in^29,32^. On the other hand, if a hollow atom is formed, the Rydberg states may remain excited and decay through Interatomic Coulombic Decay (ICD)^33,34^. As a consequence of the cascade neutralization, ions near the surface remain only partially neutralized^29^.

The population dynamics developed in^29,35^ are applied here in the context of nanostructure formation. The quantum two-state vector model^36,37^ and the micro staircase model^29^, both adapted to arbitrary collision geometry, are employed to analyze the ion neutralization process above the surface. We note that the fundamental process of electron exchange between ions and the solid surface has been described by several theoretical models, including the classical over barrier model (COB)^38,39^, the complex scaling method^40,41^, and the coupled angular mode method^42^.

The two-state vector model (TVM), used to describe the intermediate stages of Rydberg level population, represents a time-symmetric formulation of quantum mechanics. This model was originally proposed by Aharonov et al.^43^, and later developed into a closed mathematical form in^44^. Independent of these references, a Demkov-Ostrovskii type two-state vector model^45^ was introduced in surface physics in^46^, where it was applied to proton neutralization. This model was subsequently extended and systematically developed in^29,35,37,47^, focusing on the neutralization of slow, highly charged ions.

The characteristic feature of the TVM is that the state of the active electron is described by two state vectors $|\Psi _1(t)\rangle$ and $|\Psi _2(t)\rangle$, which evolve in opposite directions in time^37^. The evolution of the first state $|\Psi _1(t)\rangle$ proceeds forward in time from the initial electron state $|\mu _M\rangle$ (where the electron is primarily localized within the metal) at time $t_{in}$, and is governed by the in-Hamiltonian ${\hat{H}}_1$. On the other hand, the second state $|\Psi _2(t)\rangle$ evolves backward in time from the final electron state $|\nu _A\rangle$ (where the electron is bound to the ion) at time $t_{fin}$; the evolution is represented by the out-Hamiltonian ${\hat{H}}_2$. At the intermediate time *t* (between the initial time $t_{in}$ and the final time $t_{fin}$) the state of the active electron is expressed by two intermediate eigenstates $\mid \mu _M(R)\rangle$ and $\mid \nu _A(R)\rangle$ of the Hamiltonians ${\hat{H}}_1$ and ${\hat{H}}_2$.

**Fig. 2:**
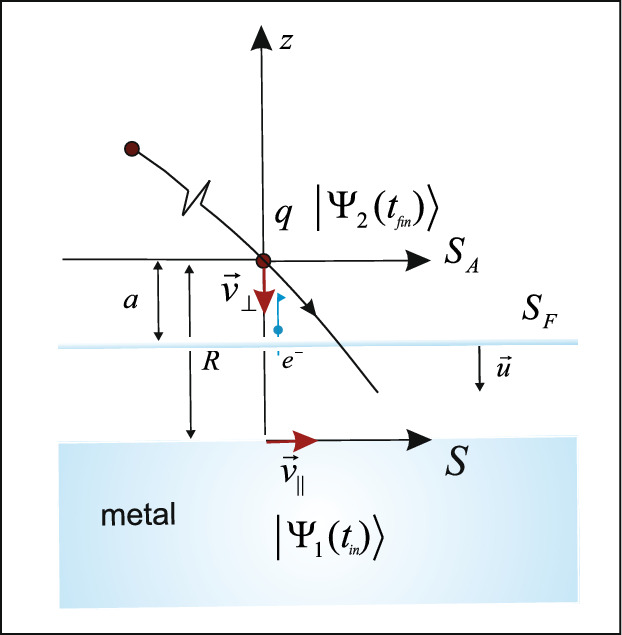
The TVM description of the neutralization process under arbitrary collision geometry. The neutralization dynamics is completely determined by the behaviour of wave functions on the Firsov plane $S_F$^29,35,37^.

The first state $|\Psi _1(t)\rangle$, defined in the coordinate system *S* moving along the target with parallel velocity $v_{\parallel }$ (see Fig. 2), is related to the state $|\Psi _1^{(0)}(t)\rangle$, which describes the state of the active electron in the rest frame $S^{}(0)$ (i.e. for $v_{\parallel }=0$) by the relation $|\Psi _1(t)\rangle =|\Psi _1^{(0)}(t)\rangle /_{\textbf{k}\rightarrow \textbf{k}^{\prime }=\textbf{k}-\textbf{v}_{\parallel }}$, where $\textbf{k}$ is the electron momentum in the solid. This relation indicates an effective change of the Fermi-Dirac distribution *f* of electron momenta in the solid, making it dependent on the magnitude and orientation of the modified vector $\textbf{k}^{\prime }$^35^. The state $|\Psi _1^{(0)}(t)\rangle$ evolves from the initial state $|\Psi _1(t_{in})\rangle =|\mu _M \rangle$, which can be expressed by the parabolic eigenstate of the in-Hamiltonian $\hat{H}_1(R)$: $|\mu _M (R)\rangle = |\gamma _M, n_{1M},m_M\rangle$. The corresponding eigenenergy is given by $E_M=-\gamma _M^2/2$ (measured from the vacuum level of the ion-surface system), where $\gamma _M$ is a continuous energy parameter. A change in the incidence angle induces a shift in the eigenenergy, such that $E_M^{\prime }=-\gamma _M^{\prime 2}/2$, where $\gamma _M^2-\gamma _M^{{\prime }^ 2}=k^{\prime 2} - k^2$.

The effect of the incidence geometry on the second state is described by the relation $|\Psi _2(t)\rangle$ $=\exp ^{i(\textbf{v}_\perp \cdot \textbf{r} -v_{\perp }^2 t/2)}$ $|\Psi _2^{(A)}(t)\rangle$, where $|\Psi _2(t)\rangle$ and $|\Psi _2^{(A)}(t)\rangle$ represent the active electron state in the system *S* (with $\textbf{r}$ being the electron position relative to *S*) and in the rest frame $S_A$ of the moving ion, respectively. The state $|\Psi _2^{(A)}(t)\rangle$ evolves toward the final Rydberg state given by $|\Psi _2(t_{fin})\rangle =|\nu _A \rangle$. The state $|\nu _A (R)\rangle = |n_A, l_A,m_A\rangle$ is a spherical eigenstate of the out-Hamiltonian $\hat{H}_2(R)$. The corresponding discrete eigenenergy is given by $E_A(R)=-\gamma _A^2(R)/2=-\tilde{\gamma }_A^2/2+(2q-1)/{4R}$, where ${\tilde{\gamma }}_A^2/2$ is the eigenenergy of the atomic Hamiltonian $\hat{H}_A$. The quantity ${\tilde{\gamma }}_A$ can be obtained from the experimentally known spectra ($E_{expt}={\tilde{E}}_A=-{\tilde{\gamma }}_A^2/2$^48^). We point out that the use of two wave functions offers a practical advantage: the main effects are characterized by different symmetries and can therefore be treated independently. Specifically, ${\hat{H}}_1$ describes a system with parabolic symmetry, while ${\hat{H}}_2$ corresponds to a system with spherical symmetry.

The population of the Rydberg states is described by the population probability $P_{\nu _A}(R)$, which depends on both the ion velocity and the incidence geometry. It is given by $P_{\nu _A}(R)=1-\exp [-\tau _{\nu _A}(R)]$, where $\tau _{\nu _A}(R)\equiv T_{\nu _A}(t)$^29^. The quantity $T_{\nu _A}(t)$ represents the transition probability and is expressed by:

1 $$\begin{aligned} T_{\nu _A}(t)=2\pi \varepsilon (R;v_\parallel , v_\perp ) T_{\nu _A}^{(0)} \frac{v_\perp }{\gamma _A|{\tilde{\beta }}|}\langle f \rangle _{\Omega _{k^\prime }} f_{\gamma }(\gamma _M^{\prime })[\gamma _M^{\prime }+\gamma _A(R)]^2 \left( 1+\frac{2{\tilde{\alpha }}}{\tilde{\beta }}\frac{1}{R}\right) R^{2\tilde{\alpha }}e^{-2\tilde{\beta }R}. \end{aligned}$$

All quantities (except $\varepsilon$) are defined for quasi-resonant electron transitions (at distance *R* we have $\gamma _M^{\prime }=\gamma _A(R)$); the quantity $\varepsilon$ accounts for the influence of nonresonant levels, where $\gamma _M^{\prime }\ne \gamma _A(R)$. The quantity $\langle f \rangle _{\Omega _{k^\prime }}$ denotes the angle-averaged Fermi-Dirac distribution of electron momenta in the solid^35^. The parameters ${\tilde{\alpha }}$ and ${\tilde{\beta }}$ are defined by $\tilde{\alpha }=q/{{\tilde{\gamma }}_A}-1/2+1/({4\gamma _M^{\prime }})$ and ${\tilde{\beta }} =\gamma _M^{\prime }+(\tilde{\gamma }_A-\gamma _M^{\prime })/2$. The explicit expressions for $T_{\nu _A}^{(0)}$ and $f_{\gamma }(\gamma _M^{\prime })$ are provided in^35^.

**Fig. 3:**
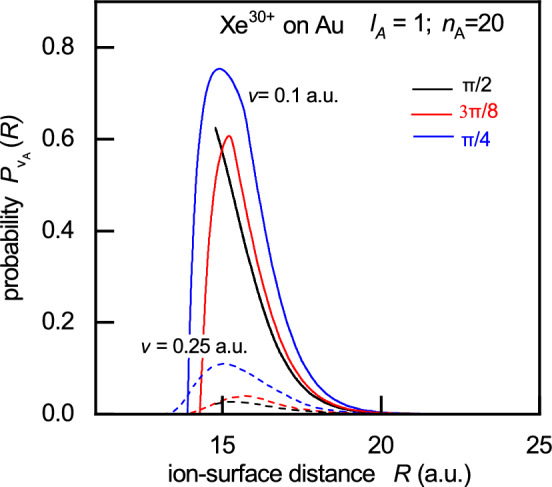
Population probability $P_{\nu _A}(R)$ versus ion-surface distance *R* for $\hbox {Xe}^{30+}$ ions impinging on a gold surface at incidence angles $\phi _{in}=\pi /2, 3\pi /8$ and $\pi /4$ with velocities $v=0.1$ a.u. (solid lines) and $v=0.25$ a.u. (dashed lines).

In Fig. 3 we present the population probability $P_{\nu _A}(R)$ of the Rydberg state for $n_A = 20, l_A=1, m_A=0$ of the $\hbox {Xe}^{30+}$ ions impinging on a gold surface, as a function of ion-surface distance *R* . The gold surface is characterized by a work function $\phi = 5$ eV, and a potential well depth $U_0=15$ eV, as described by the Sommerfield model of the metal. The incidence angles are $\Phi _{in}=\pi /2, 3\pi /8$ and $\pi /4$ and the ion velocities are $v=0.1$ a.u. (solid lines) and $v=0.25$ a.u. (dashed lines).

For incidence angles $\Phi _{in}< \pi /2$, the probabilities behave as a peak-shaped with maxima $P_{\nu _A}^{max}(R)$ at $R = R_{max}$. The initial increase in the population probability $P_{\nu _A}(R)$ as the ion approaches the surface (i.e., as *R* decreases) corresponds to pure electron capture. For this process the population rate is given by $\Gamma _{\nu _A}(R)=dP_{\nu _A}(R)/dt>0$. As *R* decreases further, i.e. for $R < R_{max}$, the population probability decreases, indicating that the reionization channel partially suppresses neutralization. In this regime, the rate becomes $\Gamma _{\nu _A}(R)=dP_{\nu _A}(R)/dt<0$^35^. This behaviour is described by the factor $\langle f \rangle _{\Omega _{k^\prime }}$ in Eq. (1).

**Fig. 4:**
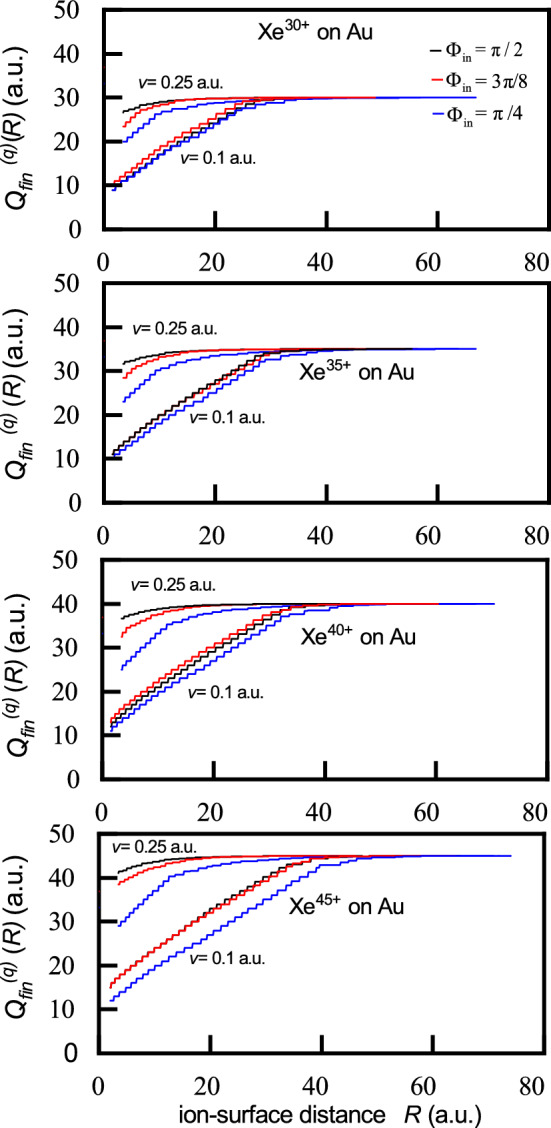
Final ion charge $Q=Q_{fin}^{(q)}(R)$ versus ion-surface distance *R* for $\hbox {Xe}^{q+}$ ions ($q=30,35,40$ and 45) impinging on a gold surface at incidence angles $\Phi _{in}=\pi /2, 3\pi /8$ and $\pi /4$ with velocities $v=0.1$ a.u. and $v=0.25$ a.u.

In the case of normal incidence ($\Phi _{in} = \pi /2$), where ($\langle f \rangle _{\Omega _{k^\prime }}\rightarrow 0$, $\gamma _M^{\prime }\rightarrow \gamma _M$) the process consists predominantly of pure population without reionization, regardless of the ion velocity. The values of $P_{\nu _A}^{max}(R)$ increase as the angle of incidence $\Phi _{in}$ decreases. This can be attributed to the reduction in the perpendicular velocity component $v_{\perp }$ and the corresponding increase in the parallel component $v_{\parallel }$ as $\Phi _{in}$ decreases (see Fig. 4 in^29^). Finally, it is observed that the maximum of the population probability for the considered Rydberg state decreases with increasing ion velocity (from $v = 0.1$ a.u. $\rightarrow v = 0.25$ a.u.).

### Final ion charge under arbitrary collision geometry

The fine structure of the cascade neutralization process, relevant in the low to moderate velocity regime, is described by the micro staircase model^29^. The most distinctive feature of this model is that each macro step consists of several micro cascades. This implies that many Rydberg levels (associated with the micro-cascades) can be populated simultaneously with low, nearly equal probabilities. Thus, the macro cascade is described as follows^29^

2 $$\begin{aligned} Q\rightarrow Q-P^{(Q,j_{\min }^{(Q)})}\rightarrow Q-[P^{(Q,j_{\min }^{(Q)})}+P^{(Q,j_{\min }^{(Q)}+1)}]...\rightarrow Q-\sum _{j=j_{\min }^{(Q)}}^{j_{\max }^{(Q)}} P^{(Q,j)}\approx Q-1. \end{aligned}$$

At intermediate ion-surface distance *R*, the ion final charge is given by

3 $$\begin{aligned} Q_{fin}^{(q)}(R)= Q_{\min }^{(q)}(R)-2\sum _{j=j_{\min }^{(Q)}}^{{\tilde{j}}_{\max }^{(Q)}}\sum _{l_A,m_A}P_{\nu _A}^{\max }. \end{aligned}$$

The quantity $Q_{\min }^{(q)}(R)$ corresponds to the last macro step and is defined as $Q_{\min }^{(q)}(R)=\{\max Q,$ $R_c^{(Q,j_{\max }^{(Q)}+1)}$ $< R\}$.

The sets of Rydberg levels with principal quantum number $n_A$, indexed by $j: n_A=n_q-j+1$, and populated during the micro cascades, are defined by the parameters $\tilde{j}_{\max }^{(Q)},j_{\min }^{(Q)}$ and $j_{\max }^{(Q)}$. The quantity ${\tilde{j}}_{\max }^{(Q)}$ is defined as ${\tilde{j}}_{\max }^{(Q)}= \{\min j, R_c^{(Q,j+1)}<R, Q=Q_{\min }^{}(q)\}$ (Eq. (19) in^29^). The minimal value of the parameter $j_{\min }^{(Q)}$ corresponds to the highest (i.e. first) Rydberg level populated within the current macro-step, associated with ion charge *Q*. It is given by $j_{\min }^{(Q)}=\{ \min j; R_c^{(Q,j)} < R_c^{(Q+1,j_{\max }^{(Q+1)}} \}$. For the first macro step, this value is $j_{\min }^{}(q)=1$, which yields $n_A=n_q-j+1=n_q$, corresponding to the highest Rydberg level populated with a significant probability. This probability exceeds the lower threshold set by $\textit{P}_{n_A}>\textit{P}_{\min }$, where $\textit{P}_{n_A}=P^{(Q,j)}=2 \sum _{l_A,m_A}P_{\nu _A}^{\max }$. The maximal value of the micro cascade index within the current macro step associated with charge *Q* is defined as $j_{\max }^{(Q)}=\{ \min j; \sum _{k=j_{min}^{(Q)}}^{j+1} \textit{P}^{(Q,k)}>1 \}$.

The quantities $P_{\nu _A}^{\max }$ represent the maxima of the population probabilities for Rydberg levels populated at the characteristic neutralization distances $R_c^N = R_c^{(Q,j)}$. In the final stage of the cascade (at $R=R_{min}$), the ion (of the initial charge *q*) reaches its final charge state $Q_{fin}^{(q)}$. The effects of both ion velocity and arbitrary geometry are expressed by the maximum value of the population probability $P_{\nu _A}^{max}$ and the corresponding neutralization distance $R_c^N$.

**Fig. 5:**
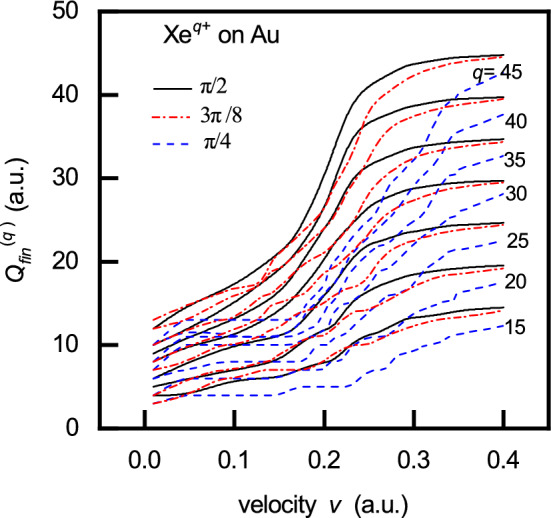
Final ion charge $Q_{fin}^{(q)}$ versus ion velocity *v* for $\hbox {Xe}^{q+}$ ions ($q=15, 20, 25, 30, 35, 40$ and 45) impinging on a gold surface at incidence angles $\Phi _{in}=\pi /2, 3\pi /8$ and $\pi /4$.

Figure 4 shows the final ion charge $Q=Q_{fin}^{(q)}(R)$ as a function of the ion-surface distance *R* for $\hbox {Xe}^{q+}$ projectiles ($q=30,35,40$ and 45) impacting a gold surface at three different incidence angles $\Phi _{in}=\pi /2, 3\pi /8$ and $\pi /4$ and at two velocities $v=0.1$ a.u. and $v=0.25$ a.u. At the low velocity ($v=0.1$ a.u.) and for lower initial ion charge states *q* (up to $q=35$), the functions $Q_{fin}^{(q)}(R)$ exhibit similar behaviour across all incidence angles. As *q* increases, the differences in $Q_{fin}^{(q)}(R)$ become more pronounced. At the higher velocity ($v=0.25$ a.u.), the final ion charges show distinct dependence on *R* that varies with the incidence angles, even for the same initial charge state *q*.

In addition, based on the relation $Q=Q_{fin}^{(q)}(R)$, the neutralization length, defined as the distance between the first and last macro step along the direction normal to the surface, can be estimated (see Fig. 1). This length will be used in calculating the deposited kinetic energy.

For the analysis of nanostructures formed on metal surfaces by the impact of highly charged ions, the most relevant quantity is the final ion charge in the very vicinity of the surface, $Q_{fin}^{(q)}=Q_{fin}^{(q)}(R_{\min })$. Figure 5 depicts the velocity dependence of $Q_{fin}^{(q)}$ for $\hbox {Xe}^{q+}$ ions interacting with a gold surface, across a range of low to moderate velocities, and for incidence angles: $\Phi _{in}=\pi /2,3\pi /8$ and $\phi =\pi /4$. The impact geometry influences the final charge state through its effect on the population probability. As the incidence angle decreases, the maximum of the population probability increases - corresponding to the last Rydberg level populated in the final macro step (see Fig. 3 in^29^)) - which results in a lower final ion charge for the same initial charge state *q* and velocity.

At very low ion velocities the micro staircase model reduces to the simple staircase model^39,49^ in which the projectile’s charge *Q* is instantaneously reduced to charge $Q-1$ at a characteristic neutralization distance $R_c^N$. This distance corresponds to the population of the Rydberg state with the critical principal quantum number $n_A=n_c(Q)$, associated with the ion charge *Q*.

### Neutralization and deposited kinetic energy under arbitrary collision geometry

Upon completion of the neutralization process, the neutralization energy is deposited into the first nanometers of the surface^50^. This process occurs very fast (within a few fs for metal targets^5,51^, resulting in an increase in the energy density in the impact region^9^. Such rapid energy deposition has been shown to induce a variety of phenomena, including the secondary electron yields^52^, secondary ion yields^53^, atomic sputtering^54^ and target-core electron excitation^55^. These effects results form charge exchange processes between ions and target atoms^5^. Furthermore, electron excitations triggered by the cascade neutralization occurring above and partially within the target represent the primary mechanism by which the neutralization energy contributes to surface modification. This mechanism marks the initial stage of surface nanostructuring^25,28^.

The neutralization energy is calculated as the difference between the potential energy $W_{q,pot}$ of the ion before the onset of the neutralization process and the potential energy $W_{Q_{fin},pot}^{(q)}$ of the ion after the neutralization is completed^27,30,56^:

4 $$\begin{aligned} W^{(q)}= W_{q,pot}-W_{Q_{fin},pot}^{(q)}. \end{aligned}$$

The initial potential energy $W_{q,pot}$ is determined using energy spectra data, i.e. by summation of the ionization energies of the ground state of HCI over the range from $Q=q$ to $Q=1$^48^. It is worth mentioning here that for very low ion velocities, where $Q_{fin}^{(q)}\ll q$, the neutralization energy is approximately equal to the full potential energy $W^{(q)}\approx W_{q,pot}$. At moderate ion velocities, however, the neutralization process is incomplete. As a result, the neutralization energy is lower than the full potential energy.

Once the neutralization process is completed in front of the target, the ion in its final charge state $Q_{{fin}}^{(q)}$, enters the solid and begins to slow down. Energy deposition into the target causes disruptions in both the atomic and electronic subsystems of the material. This occurs through two primary mechanisms: elastic collisions with atomic nuclei (nuclear stopping power), and inelastic collisions with target electrons (electronic stopping power). The kinetic energy transferred from the projectile to an atom during a single ion-atom collision can exceed the lattice binding energy, causing the atom to be permanently displaced from its lattice site. This atom may, in turns displace additional atoms in subsequent recoil events. Higher-order generations of recoils can be produced in a similar manner, resulting in a collision cascade. Meanwhile, the electronic stopping power leads to ionization, the generation of electron–hole pairs, and plasmon formations^51^. In the velocity regime studied here, nuclear (elastic) energy loss dominates over electronic energy loss and is sufficient to define the total deposited energy in combination with the neutralization energy. Thus, electronic stopping power has rather little direct effect on damage production in the materials studied here^3,26,27^, $dE_e/dx\approx 0.1 dE_n/dx$^3^. Both the neutralization energy and the deposited kinetic energy (the portion of ion’s kinetic energy transferred to the solid through elastic collisions with atomic nuclei are essential for surface modification^4,5,27,57–59^. However, one of these contributions typically dominates, thereby governing the type of nanostructure that forms. This balance is determined by the critical ion velocity, at which these two energy contributions become equal. Depending on whether the neutralization energy or deposited kinetic energy is the principal energy source, either hillocks or craters will appear, respectively. Finally, the contribution of ion neutralization occurring below the surface has a negligible effect on the total deposited energy^25^.

The kinetic energy loss is considered within the charge-dependent model of ion-metal surface interaction. It is worth noting that the first theoretical prediction of the charge dependence of energy loss was introduced by Biersack^31,60^. Experimental results^61,62^ revealed a significant enhancement in energy loss compared to the SRIM values^63^. Furthermore, in^64^, the authors developed a detailed charge-dependent model for kinetic energy transfer during HCI interactions with carbon, which showed good agreement with experimental data^61,62^. Charge-dependent kinetic energy loss was also observed in^65^ and was theoretically explained using the statistical atom model^31^. A more accurate model for calculating kinetic energy loss was later proposed in^66^, incorporating a time-dependent interatomic potential that also accounts for the excitation of the target atom.

To calculate the kinetic energy loss we employ a charge-dependent interaction potential model. To construct the interaction potential, we take into account that the projectile final charge, $Q=Q_{fin}^{(q)}$, depends on the initial charge *q*, the velocity and the angle of incidence. At an internuclear distance *r*, the interaction potential is given by^28,31^:

5 $$\begin{aligned} V_{int}(r)=\frac{(Z_1-Q_{fin}^{(q)}))Z_2}{r}\phi \left( \frac{r}{a_u}\right) +\frac{Q_{fin}^{(q)}) Z_2}{r}\phi \left( \frac{r}{a_s}\right) , \end{aligned}$$

where $Z_1$ and $Z_2$ are the nuclear charges of the projectile and the target atom, respectively. The screening lengths for the interaction of the atom of nuclear charge $Z_1-Q_{fin}^{(q)}$ with the target atom and the point charge $Q_{fin}^{(q)}$ with the target atom, are $a_u=0.8853/\left( (Z_1-Q_{fin}^{(q)}))^{0.23}+Z_2^{0.23})\right)$ and $a_s=0.8853/Z_2^{1/3}$, respectively. We employ the standard analytical form of the Ziegler–Biersack–Littmark screening function $\phi \left( {r}/{a}\right)$ $=\sum _i c_i\exp (-d_ir/a)$, where $c_i$ and $d_i$ ($i\in [1,4]$) are constants given in^67^.

**Fig. 6:**
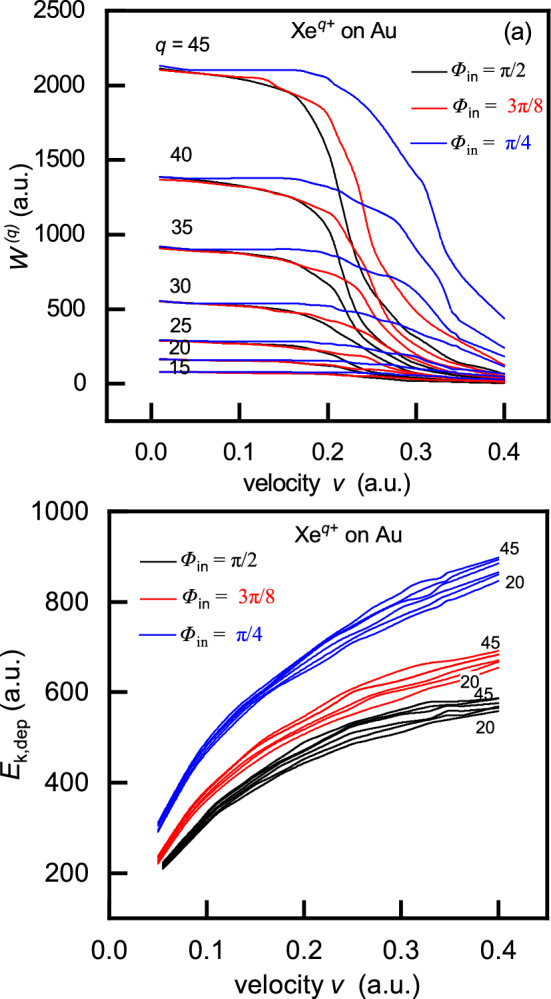
(**a**) Neutralization energy $W^{(q)}$ and (**b**) deposited kinetic energy $E_{k,dep}$ as functions of ion velocity for $\hbox {Xe}^{q+}$ ions ($q=20, 25, 30, 35, 40$ and 45) impinging on a gold surface($\phi =5$ eV) at incidence angles $\Phi _{in}=\pi /2,3\pi /8$ and $\pi /4$.

According to our model, nanostructure formation is considered a surface effect. This means that the neutralization above the surface and the energy deposition below it occur within a near-surface of approximately equal extent, see^25^. The interaction depth $\Delta {\tilde{x}}$ below the surface, measured along the direction perpendicular to the surface, is related to the interaction length $\Delta x$ along the ion trajectory by the expression $\Delta {\tilde{x}} = \Delta x \sin {\Phi _{in}}$, see Fig. 1. The interaction depth $\Delta {\tilde{x}}$ is typically on the order of several atomic layers^25^.

We calculate the kinetic energy transferred in collisions between the projectile and target atoms within the active volume ${\tilde{V}}$^25,68^. Within our model, the active volume is assumed to have a shape of a cylinder whose diameter *D* coincides with that of the formed nanostructure: ${\tilde{V}}= (D^2/4) \pi \Delta {\tilde{x}}$, see Fig. 1 The contribution of the nuclear stopping to the deposited kinetic energy is calculated using the nuclear stopping cross section, given by $S_n= 2 \pi \int _{0}^{\rho _{max}} \rho T d\rho$^69^, where $\rho$ is the impact parameter^28^. The energy loss per unit path length is then expressed as $dE_n/dx=nS_n$, with *n* denoting the atomic density of the target. The quantity *T* represents the energy transferred in a single elastic scattering event, across the surface area determined by the impact parameter $\rho$^28,31,69^. It is related to the maximal value $T_{max}$ by the relation $T=T_{max}\sin ^2(\theta (Q,\rho )_{CMS}/2)$, where $\theta (Q,\rho )_{CMS}$ is the scattering angle in the center of mass system (CMS)^28^ given by:

6 $$\begin{aligned} \theta (Q,\rho )_{cms} = \pi - 2 \int_{r_{min}}^{\infty } dr \frac{\rho }{r^2} \left[ 1-\frac{V_{{int}}(r)}{E_{{cms}}}-\frac{\rho ^2}{r^2} \right] ^{-1/2}. \end{aligned}$$

The influence of the incidence angle on the deposited kinetic energy arises through its effect on the final ion charge $Q=Q_{fin}^{(q)}$ and on the interaction depth $\Delta {\tilde{x}}$, which is measured perpendicular to the surface, see Fig. 1.

In Fig. 6, we present the neutralization energy $W^{(q)}$ and the deposited kinetic energy $E_{k,dep}$ as functions of the ion velocity *v*, for the interaction of slow $\hbox {Xe}^{q+}$ ions (with charge states $q\in [20,...,45]$) with a gold target. The work function of gold is $\phi =5$ eV and the atomic density is $n=8.7\cdot 10^{-3}$ $\hbox {cm}^{-3}$^70^. Finally, the deposited kinetic energy due to nuclear collisions is given by $E_{k,dep}= n S_n \Delta x$, where the length $\Delta x$ is connected to the interaction depth $\Delta {\tilde{x}}$ through incidence angle, see Fig. 1. The interaction depth is approximately $\Delta {\tilde{x}} \approx 5 {\bar{c}}$^25,28^, where ${\bar{c}}$ is the mean lattice constant; for the gold target, ${\bar{c}}=0.407$ nm ($\Delta x=38.5$ a.u.). The estimation of the interaction depth is given in Section “Second step of the cohesive energy model (CEM II)”.

As shown in Fig. 6, the theory predicts that both the neutralization energy and the deposited kinetic energy increase, with decreasing angle of incidence, for a given ion charge *q* and velocity *v*. This behaviour arises from the dependence of the final ion charge on the angle of incidence (see Fig. 4).

## Nanostructure shape under arbitrary collision geometry

### Second step of the cohesive energy model (CEM II)

Within the framework of the second step of the cohesive energy model (CEM II)^25^, surface modification, manifested as nanostructure formation (hillocks and craters), is interpreted as a direct consequence of changes in cohesive energy induced by the total deposited energy. Cohesive energy is considered as a fundamental quantity characterizing metallic bonding. The essential part of metallic bonding is a heavy overlap between the individual valence electron wave functions, resulting in a high degree of delocalization of valence electrons.

By definition, the initial and final cohesive energies of the solid are given by $E_{in,coh}=E_{in,solid}-E_{atom}$ and $E_{fin,coh}=E_{fin,solid}-E_{atom}$, where quantities $E_{in,solid}$ and $E_{fin,solid}$ denote the total energies of the unperturbed and perturbed target, respectively (both have negative values). In the second CEM II step, the total energy of the crystal is expressed as a function of a set of structural parameters $\{R\}$ defining the lattice structure. Accordingly: $E_{in,solid}=E_{solid}\{R_{0,in}\}$ and $E_{fin,solid}=E_{solid}\{R_{0,fin}\}$, where $\{R_{0,in}\}$ and $\{R_{0,fin}\}$ are the structural parameters corresponding to the crystal lattice in its equilibrium configuration (unperturbed target) and the modified lattice structure following nanostructure formation, respectively. The energy $E_{atom}$ represents the collective energy of isolated atoms and can be expressed as $E_{atom}= E_{solid}(\{R_0\}\rightarrow \infty )$, i.e. the total energy of the system when the interatomic separations are taken to infinity^68^.

Following the core energy balance principle of the CEM, the total deposited energy satisfies the relation^25^:

7 $$\begin{aligned} E_{tot,dep}= E_{fin,coh} - E_{in,coh} + \Delta E, \end{aligned}$$

where $\Delta E$ accounts for additional contributions, primarily the energy carried away by ejected atoms. In the case of hillock formation, this contribution is small and can be neglected compared to the total deposited energy.

The main idea of the CEM II step is to analyse the size of the formed nanostructure using the concept of cohesive energy. In this article, we focus exclusively on the shape of the nanostructure. The detailed formulation of the CEM II step is provided in^25,68^. A brief overview is presented here, which is necessary for estimating the interaction depth over which the total energy is deposited. This enables us to establish a relation between the interaction depth and experimental observables such as hillock height and crater depth, using a self-consistent approach.

As already presented in^25,68^, the energy balance can be rewritten in a more convenient form $E_{tot,dep}$ $= E_{c0} - E_c$ $+ \Delta E$. Here, $E_{c0}=$ $-E_{in,coh}$ and $E_{c}=$ $-E_{fin,coh}$ represent the initial and final cohesive energies of the unperturbed (of the initial, active volume ${\tilde{V}}$) and perturbed target (of the final volume $V_{fin}$), respectively. In the case of hillock formation, the final volume is $V_{fin}=V+V_{hill}$, while for crater formation, it is $V_{fin}=V-V_{crat}$. Both hillock and crater are modelled as rotational paraboloids of diameter *D* and height *h* or *d* (hillock’s height *h* and crater’s depth *d*, respectively) with a volumes given by $V_{hill}=1/2 (D^2/4)\pi h$ and $V_{cr}=1/2 (D^2/4)\pi d$^25,68^.

The initial cohesive energy is the same for both types of nanostructure and is given by $E_{c0}=n_0U_0V$, where $n_0$ and $U_0$ are the initial atomic density and absolute value of cohesive energy per atom (experimental value), respectively. For a gold target, these values are $n_0=0.0589 \text {{\text{\AA }}}^{-3} = 8.7\cdot 10^{-3}$ a.u. and $U_0=3.93$ eV$=0.144$ a.u.^70^

The final cohesive energy for hillock formation is $E_c^{hill}=nVU+n_{hill}U_{hill}V_{hill}$ and for crater formation $E_c^{crat}={\tilde{n}} (V-V_{crat}){\tilde{U}}$, where $n_{hill}, U_{hill}$ and $V_{hill}$ characterize the hillock and $\tilde{n},V_{cr}$ and ${\tilde{U}}$ characterize the crater.

Following the procedure outlined in^25,68^, we obtain expressions for the total deposited energy in both cases. We start by applying particle number conservation. For the hillock, it is expressed by $n_0V$ $=nV+n_{hill} V_{hill}$ $+\Delta N_h$, while for the crater it is given by $n_0 V$ $= {\tilde{n}}(V-V_{crat})$ $+\Delta N_c$. Here $\Delta N_{h/c}$ is the number of ejected atoms. In particular, $\Delta N_c=n_0V_{crat}$. For hillock production, the total deposited energy becomes $E_{tot,dep}^{hill} =n_0 U_0 V\left( 1-(U/U_0)(1-\chi )+\Delta E/(n_0U_0V)\right)$, which simplifies to $E_{tot,dep}^{hill} =n_0 U_0 V\left( 1-(U/U_0)(1-\chi )\right)$, since $\Delta E/(n_0U_0V)$ is negligible. In the case of crater formation, the total deposited energy is given by $E_{tot,dep}^{crat} =n_0 \left( U_0 - {\tilde{U}} \right) V + \Delta N_c \left( {\tilde{U}} +E_{kat}\right)$, where $E_{kat}$ is the kinetic energy of an ejected atom, estimated based on experimental conditions^5,25,71^.

The deposited kinetic energy can also be written as $$E_{tot,dep}^{hill/crat}$$ $$=\kappa _c^{hill/crat}$$ $$E_{c0}$$ with cohesive energy parameters $$\kappa _c^{hill/crat}$$. In the case of hillock, it is written as $$\kappa _c^{hill} = 1-(U/U_0)(1-\chi )$$, while for crater formation, it takes the form $$\kappa _c^{crat}$$ $$= 1-(\tilde{U}/U_0)( 1-\Delta N_c/(n_0V))$$ $$+\Delta N_cE_{kat}/E_{c0}$$. The parameter $$\chi$$ is given by $$\chi =$$ $$(n_{hill}/n_0)(V_{hill}/V)(1-U_{hill}/U)$$ $$+\Delta N_h/n_0V$$, which simplifies to $$\chi =$$ $$(n_{hill}/n_0)($$ $$V_{hill}/V)$$ $$(1-U_{hill}/U)$$, since $$\Delta N_h/n_0V\approx 0$$. Using the uniform density approximation, $$n\approx n_{hill}$$ and $$U \approx U_{hill}$$, and noting that the atomic densities in the modified volumes may slightly deviate from those of the unperturbed target^72^, we get $$\chi =0$$. Thus, the final expressions for the cohesive energy parameters are $$\kappa _c^{hill} = 1-(U/U_0)$$ for hillock formation and $$\kappa _c^{crat}=1-\tilde{U}/U_0(1-V_{cr}/V)$$, for crater formation. The latter expression simplifies to $$\kappa _c^{crat}=1-{\tilde{U}}/U_0(1-d/2\Delta {\tilde{x}} )$$^4,5,68,71^.

To analyse surface nanostructure creation, we employ two parameters that describe the extent of surface modification. The first is the cohesive energy parameter $\kappa _c^{hill/crat}$, already defined, which characterize the nanostructure type. The second is a parameter that quantifies the degree of crystal structure modification, denoted as $a^{\star }$. This parameter is related to the changes in cohesive energy per atom through the relation $(U^{\star }/U_0)= (1+a^{\star }) e^{- a^{\star }}$, where $U^{\star }=U$ for hillock formation and $U^{\star }={\tilde{U}}$ for crater formation. The parameter $a^{\star }$ is defined in terms of variations in interatomic distances and provides a physically meaningful measure of crystallographic changes induced by ion irradiation: $a^{\star }=\eta (\beta -1)$, with $\eta =a_0/r$ and $\beta =a/a_0$, where $a_0$ and *a* denote the initial and final closest interatomic distances in the unperturbed and perturbed target, respectively, and *r* is the atomic radius. This approach allows us to establish a simple relation between the changes in atomic density and interatomic spacing, $n_0/n=(a/a_0)^3$, which is characteristic of the simple cubic structure^25,68^.

From this expression and from the law of particle number conservation, we derive the relation between the variation in interatomic distances and hillock height, which enables the estimation of the interaction depth $(a/a_0)^3=1+h/2\Delta {\tilde{x}}$^68^. This relation allows us to determine the maximum change in the crystallographic structure of the target $a/a_0$, that leads to the formation of a specific nanostructure. Thus, the disruption of the regular arrangement of atoms in the crystal lattice can manifest as an increase in interatomic spacing ($a_0\rightarrow a$), leading to bond weakening. According to the experiment in^72^ the maximum increase in interatomic spacing is up to $5\%$ which induces a corresponding decrease in atomic density of up to $10\%$. In the case of hillock formation, such changes in interatomic distances can be regarded as a solid-liquid phase transition (melting) within the metal^59^, implying a transformation from a crystalline to an amorphous metallic structure^72^.

The depth over which the total energy is deposited can only be very roughly estimated. Here, we estimate the depth that is consistent with available experimental data. Based on the above expressions and in correlation with experimental findings^68,72^, even though many parameters remain unknown due to a lack of measurements, it is possible to predict the interaction depth sufficient to induce structural changes associated with a slightly altered atomic density relative to the unperturbed target. In the case of hillock formation, this interaction length satisfies the relation $\Delta {\tilde{x}} \le 3h$^68^. In the near-surface region ($\Delta {\tilde{x}} \le h$), both the neutralization energy $W^{(q)}$ and the deposited kinetic energy $E_{k,dep}$ contribute additively^4,58,59,73^. In the first few atomic layers, the dominant contribution arises from the neutralization energy, while at greater depths, the deposited kinetic energy becomes the primary energy source^24,59^.

In the case of crater formation, the process is significantly different. Namely, due to the higher ion velocities required for crater creation, the target experiences a strong perturbation that leads to melting and changes in atomic density. At the final stage of the process, the target recrystallizes, so no permanent structural changes remain and no retardation related perturbation is observed. The similar recrystallization process of the target after SHI irradiation is presented in^74^. In the volume ${\tilde{V}}-V_{cr}$ both atomic density and cohesive energy per atom remain unchanged, ${\tilde{n}}\approx n_0$ and ${\tilde{U}}\approx U_0$. This fact allows us to determine the cohesive energy parameter and estimate the interaction depth required for energy deposition sufficient to produce a nanocrater.

Based on these estimations, the final form of the cohesive energy parameter is given by $\kappa _c^{crat}=(d/2 \Delta {\tilde{x}})$ and according to the experimental findings^4^, the minimal depth for energy deposition in the case of crater formation is $\Delta {\tilde{x}}\ge 2d$. We therefore calculate the kinetic energy loss within an interaction depth that is smaller than both the equilibration length and the mean projected range, but still consistent with the region where the formation of the nanostructure occurs and with the experimental data for both hillock and crater formation^3,4^, where $2d\approx h/2$.

Thus, the most relevant region that satisfies the necessary conditions for nanostructure formation and provides a unified picture of the process is $\Delta {\tilde{x}} \approx 2d \approx 2$nm. In this region, as mentioned earlier, both the neutralization energy and deposited kinetic energy contribute in an additive way. This length can also be expressed through lattice structure characteristics, namely the lattice constant: $\Delta {\tilde{x}} \approx 5{\overline{c}}$, where ${\overline{c}}$ is the mean lattice constant ${\overline{c}}=407$nm$=7.7$ a.u.

These findings can be correlated with the analysis presented in^59^, where the authors demonstrated the additivity of potential energy and electronic stopping power, expressed by the formula $d/F=E_p/S_e$, where *d* is the depth at which the fraction $F*E_p$ of the potential energy is deposited. To derive this relation, they considered two extreme cases: the potential energy threshold ($F*E_p$) for very low velocities ($S_e=0$), and the electronic stopping power $S_e$ for which $E_p=0$. They also showed that this depth can vary with the potential energy.

In our case, the interaction depth can be estimated using extreme values for the neutralization energy (at low ion velocity) and the nuclear stopping power (at the highest velocity considered) across all charge states examined here ($q=20,25,30,35,40,45$). Following a similar approach and applying the expression $d/F=W_{q,pot}/S_n$ we obtain interaction depths of approximately $\Delta {\tilde{x}} \approx 0.5, 0.9, 1.6, 2.7, 4.1, 6.2$ nm for the respective charge states. In our model, factor *F* is velocity dependent as presented in Fig. 3 in^28^. The result shows that the interaction depth increases with the potential energy (charge state). Accordingly, the mean interaction length is approximately $\Delta {\tilde{x}} \approx 2.6$ nm. The obtained average value corresponds closely to the length estimated using a self-consistent procedure discussed earlier.

As concluded in^59,68^, the same result follows from the brief overview of the CEM II model presented here. An important aspect for testing the model would be direct measurements of the depth of the molten phase - namely, the region responsible for inducing hillock or crater formation (i.e. the interaction depth).

### Mechanism of nanostructure creation

The dominant energy contribution determines the type of nanostructure formed on the metal surface. Specifically, the appearance of hillocks is associated with a regime in which the neutralization energy dominates, whereas craters formation is driven by the dominance of kinetic energy^4,28,75^. The transition between these two regimes is governed by the critical ionic velocity, defined by the condition $W^{(q)}(v_c)=E_{k,dep}(v_c)$. This critical velocity delineates two distinct regimes: for $v < v_c$, hillock formation is favored, while for $v> v_c$, crater formation becomes the dominant surface modification mechanism.

Threshold behaviour has also been reported for insulating materials. For example, the formation of hillock-like structures on $\hbox {CaF}_2$ and LiF surfaces under the impact of $\hbox {Xe}^{q+}$ ions has been shown to require a minimum potential energy^6,76,77^. Specifically, hillocks on the $\hbox {CaF}_2$ surface appear only when the ion’s potential energy exceeds a critical threshold depending on the projectile’s kinetic energy. A similar threshold has been identified in the formation of pit-like structure on the KBr surface, as illustrated in the corresponding phase diagram^77^. Moreover, the study presented in^59^ highlights the experimental significance of the interplay between deposited kinetic energy and released neutralization energy. Their results indicate that the formation of specific nanostructures depends on the combination of these two energy contributions, a relationship effectively visualised in the phase diagram.

The formation of hillock-like structures can be attributed to a sequence of interactions occurring as a highly charged ion approaches and penetrates the solid surface^25,28^. Initially, the ion induces a rearrangement of electron density and the formation of surface charges, leading to solid polarization. This polarization is characterized by the image force, as described within the two state vector model (TVM). Simultaneously, electrons tunnel through the potential barrier and populate high lying Rydberg states. These excited states decay to the (almost) ground states through radiative processes and when the ion approaches the surface-via Auger-type processes accompanied by secondary electron emission. These processes are governed by the population dynamics outlined in^29,32^. On the other hand, if a hollow atom is formed, the Rydberg states may remain excited and decay through Interatomic Coulombic Decay (ICD)^33,34^). For hillock formation, the ion velocity is relatively low, ensuring a sufficiently long interaction time for multiple electron captures. Simultaneously, surface polarization and ion neutralization processes lead to the deposition of neutralization energy into the solid, altering the electron density and weakening metallic bonding. This energy input results in an increase in the cohesive energy, destabilizing the local lattice structure, initiating the first step of surface modification (see Section 2.3). As the projectile moves below the surface, it undergoes elastic collisions with target atoms, transferring its momentum. Although this process slightly contributes to the total energy deposition, it influences the atomic displacement toward the surface. However, these atoms do not acquire sufficient energy to overcome the surface binding energy and remain attached to the target.

According to the CEM, crater formation primarily results from interactions between the projectile and atoms below the surface^25,28^. In this regime, the ion velocity is high, resulting in a short interaction time while it is above the surface. Consequently, only a small number of electrons are captured into the Rydberg states. This leads to a minimal amount of neutralization energy being deposited into the surface, making a small contribution to the alteration of cohesive energy. The dominant contribution in this regime arises from the deposited kinetic energy. As the ion, in its final charge state, penetrates the solid, it undergoes a series of elastic collisions with target atoms, causing a collision cascade. As a result of this cascade, a substantial momentum is transferred to the target atoms, leading to the breaking of metallic bonds and the ejection of a large number of atoms from the surface. The effect of atomic displacements give rise to crater formation. In the final stage of the process, the metallic bonds within the remaining active volume are restored as the system relaxes toward a new equilibrium configuration. The similar recrystallization process of the target after ion irradiation is presented in^74^.

## Critical ion velocity under arbitrary collision geometry

**Fig. 7:**
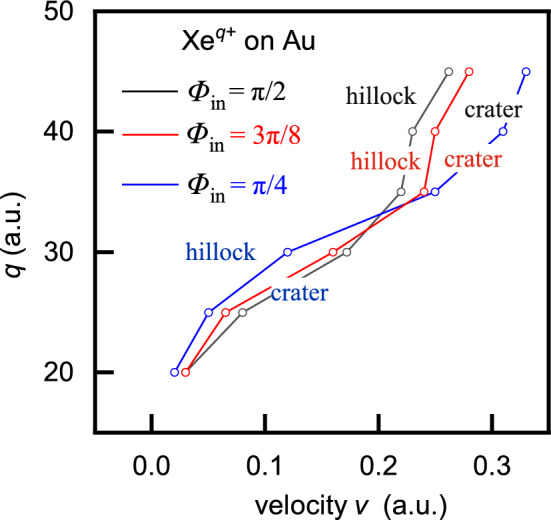
Critical ion velocities for $\hbox {Xe}^{q+}$ ions impinging on a gold surface at incidence angles $\Phi _{in}=\pi /2,3\pi /8$ and $\Phi _{in}=\pi /4$.

Within the framework of the cohesive energy model (CEM), the type of nanostructure formed is primarily governed by the critical ion velocity, which distinguishes the dominant energy source responsible for surface modification (see Section 3). The calculated critical ion velocities for $\hbox {Xe}^{q+}$ ions interacting with a gold surface at three incidence angles: $\Phi _{in}=\pi /2,3\pi /8$, and $\Phi _{in}=\pi /4$ are presented in Fig. 7 and Table1. The diagram clearly delineates the regions where hillock-like structures ($v<v_c$) and crater-like structures ($v>v_c$) predominantly form in the studied HCI-metal system. However, when analyzing the effect of the incidence angle, this dependence cannot be considered straightforward. The total deposited energy, as well as the interplay between the neutralization energy and kinetic energy loss, play an important role in determining the type of nanostructure formed.

**Table 1:** Critical velocities $v_c$ for surface modification induced by $\hbox {Xe}^{q+}$ ions impacting a gold surface at incidence angles $\Phi _{in}=\pi /2,3\pi /8$ and $\Phi _{in}=\pi /4$.

*q*	20	25	30	35	40	45
$$\phi _{in}=\pi /2$$						
$v_c$ (a.u.)	0.03	0.08	0.17	0.22	0.23	0.26
$$\phi _{in}=3\pi /8$$						
$v_c$ (a.u.)	0.03	0.065	0.16	0.24	0.25	0.28
$$\phi _{in}=\pi /4$$						
$v_c$ (a.u.)	0.02	0.05	0.12	0.25	0.31	0.33

**Fig. 8:**
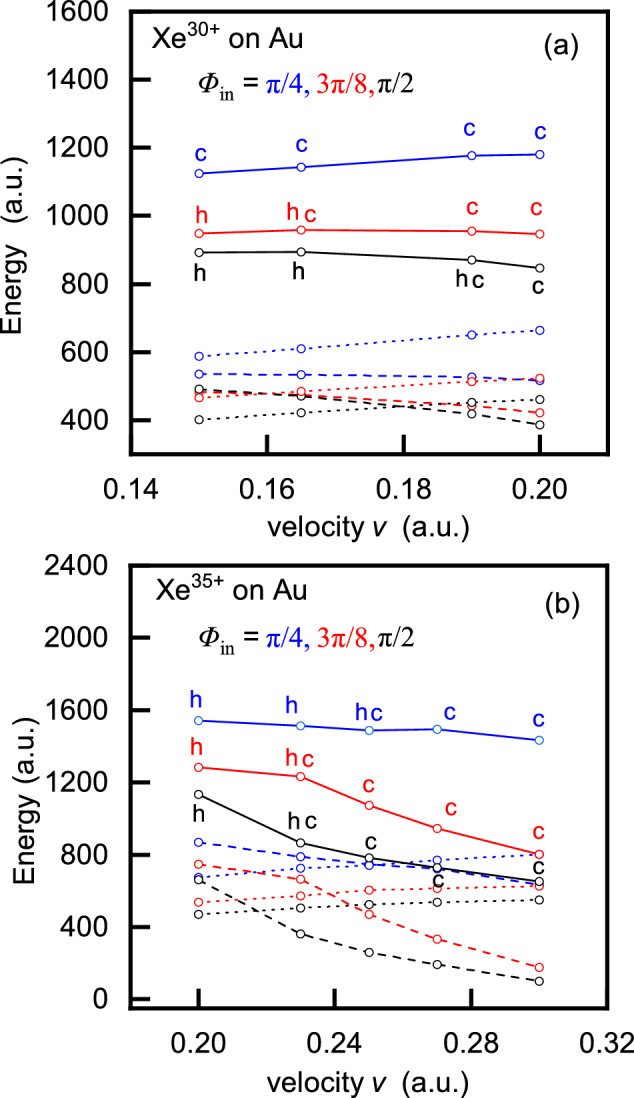
Total deposited energy (solid lines), neutralization energy (dashed lines) and kinetic energy loss (dotted lines) versus ion velocity for (**a**) $\hbox {Xe}^{30+}$ ions (**b**) $\hbox {Xe}^{35+}$ ions impinging on a gold surface at incidence angles $\Phi _{in}=\pi /2,3\pi /8$ and $\Phi _{in}=\pi /4$.

Two distinct energy regimes can be observed in Fig. 7. At low velocities, the critical velocity decreases with decreasing incidence angle. For example, at velocities up to $v=0.19$ a.u., craters are expected at $\Phi _{in}=\pi /4$, while hillocks dominate at normal incidence. This observation is consistent with Fig. 8, and also aligns with experimental electron yield data - higher yields correlate with a higher probability of crater formation. On the other hand, at higher velocities and charge state (e.g., $q=35$), the behavior is reversed: for the same ion velocity and charge state, craters are predicted at normal incidence, while hillocks may form at $\Phi _{in}=\pi /4$, according to the critical velocity definition (see Fig. 7). However, incorporating the influence of the incidence angle on the total deposited energy and on the contributions of its components ($W^{(q)}$ and $E_{k,dep}$) provides a more comprehensive understanding of the process.

For example, for $q=30$ and $\Phi _{in}=\pi /2$, hillock like structures (h) are formed at velocities up to $v=0.18$ a.u., see Fig. 8a. At slightly higher velocities, around $v=0.19$ a.u. both structures (hc) may form, as the contributions of the neutralization energy and kinetic energy loss are comparable. At even higher velocities, craters (c) become dominant. For the same charge state and $\Phi _{in}=3\pi /8$ hillocks are expected up to 0.16 a.u., while for velocities up to 0.17 a.u. both structure (hc) may form. At velocities above 0.18 a.u. only craters are likely to form. In the case of $\Phi _{in}=\pi /4$, craters (c) are predicted for all velocities within the considered range. A similar analysis can be carried out for $q=35$ and all incidence angles, as shown in Fig. 8b.

Analyzing Fig. 8, one can estimate the energy threshold $E_{th}$ for the formation of a specific nanostructure. For example, for $q=30$ at normal incidence, this threshold is approximately $E_{th}\approx 900$ a.u. For $q=35$, the threshold energy are approximately $E_{th} \approx 1000, 1200,$ and 1500 a.u for incidence angles $\Phi _{in}=\pi /2, 3\pi /8$ and $\pi /4$, respectively. This suggests that around these threshold values, both hillocks and craters can form, as the contributions from the neutralization energy and kinetic energy loss are nearly equal.

Figure 8 shows that both the total deposited energy and kinetic energy loss increase with decreasing incidence angle. This suggests that lower incidence angles lead to higher energy deposition, thereby increasing the likelihood of crater formation, as supported by experimental electron yield data.

At larger incidence angles (i.e. closer to normal incidence), the ion approaches the surface more directly, resulting in a shorter ion-surface interaction time. In this case, the ion also penetrates deeper into the material, which leads to a reduction in surface emission. Conversely, at lower incidence angles, the interaction time is longer, enabling more efficient energy deposition through neutralization process, and thus an increase in neutralization energy. Since the ion remains close to the surface and a large portion of energy is deposited in the topmost surface layers, the energy density increases, thereby enhancing the likelihood of crater formation. Moreover, as the ion enters the target at a low angle, its path length within the surface layers increases, resulting in a greater deposited kinetic energy.

Therefore, by tuning both the ion velocity and the incidence angle, specific nanostructures can be deliberately formed, as illustrated in Figs. 7 and 8. It is important to note, however, that the existing experimental data correspond only to normal incidence. Nevertheless, our theoretical predictions are in good agreement with the experimental findings reported in^3–5^ as discussed in^25^. To date, the angular dependence of total electron yields represents the only available experimental evidence on the effect of the incidence angle in HCI-metal interactions. Since no experimental data exist on how the incidence angle influences nanostructure formation in HCI-metal surface interactions, the main aim of this article is to provide theoretical insight into this open issue.

## Concluding remarks

In the present study, we extended the cohesive energy model to analyse the incidence geometry effect on nanostructure formation on a gold surface induced by the impact of slow, highly charged $\hbox {Xe}^{q+}$ ions at low to moderate velocities. In the first step of the model, we considered the total energy deposition, which consists of the neutralization energy and the deposited kinetic energy. We begin by examining the neutralization process that occurs above the surface. By applying the modified two-state vector model (TVM) and the micro staircase model to arbitrary collision geometries, we calculated the population probabilities. The influence of the incidence angle on the first wave function $|\Psi _1(t)\rangle$ is included through an energy shift that depends on the parallel velocity component $v_\parallel$, whereas its influence on the second wave function $|\Psi _2(t)\rangle$ is incorporated through Galilean invariance, which introduces a translation factor determined by the perpendicular velocity component $v_\perp$^29,35^. The result of the process above the surface is the ion at the final charge state in the very vicinity of the surface, along with the corresponding neutralization energy deposited into the solid. Both the final ion charge (essential for determining the deposited energies) and the neutralization energy are affected by ion velocity and the incidence angle through their influence on the maximum population probability. As the incidence angle decreases, population probabilities increase, leading to lower final ion charges and greater neutralization energies.

The outcome of the process below the surface is the kinetic energy deposition, calculated by using a modified interaction potential model. The resulting values of the neutralization energy $W^{(q)}$ and deposited kinetic energy $E_{k,dep}$, expressed as functions of ion charge, velocity and incidence angle, provide a key insight into the energy dominance within the metal surface, governing the nanostructure formation. The incidence angle significantly influences both energies: for a given ion charge and velocity, decreasing the angle increases both the neutralization energy and the deposited kinetic energy, thereby enhancing the total energy delivered to the surface. At low incidence angles, the deposited kinetic energy surpasses the neutralization energy, favoring the crater formation. In contrast, at higher incidence angles, the total deposited energy decreases, resulting in hillock formation over a larger velocity range compared to lower incidence angle. The concept of a critical ion velocity, introduced to characterize the dominant energy channel in surface modification, also depends on the incidence angle. Accordingly, the type of nano-sized object formed is governed by the collision geometry as well as the ion charge and velocity. Thus, the model predicts that specific nanostructure can be deliberately formed by tuning the ion velocity and the incidence angle.

In the second step of the model (CEM II), we introduced the concept of cohesive energy as a key quantity characterizing metallic bonding. The central idea of the CEM II step is that surface modification can be understood as a consequence of changes in cohesive energy resulting from the total deposited energy.

A few additional remarks may be relevant for future theoretical and experimental studies of surface nanostructures.

The results regarding the influence of collision geometry on nanostructure formation can be directly extended to other ion-metal combinations. Therefore, systematic experiments with different ion-metallic surface systems are essential for further development of the model. Furthermore, the diameters of nanostructures formed under different ion-surface combinations and collision geometries can also be analyzed within the framework of the cohesive energy model.

## Data Availability

This manuscript has no associated data or the data will not be deposited. All data needed to evaluate the conclusions in this work are present in the paper. Additional raw data are available from the corresponding author upon reasonable request.
